# Comparison between compositional data analysis and principal component analysis for identifying dietary patterns associated with hyperuricemia

**DOI:** 10.3389/fnut.2025.1582674

**Published:** 2025-07-16

**Authors:** Junkang Zhao, Yajie Zhao, Jiannan Han, Yixuan Zhao, Sumiao Liu, Zhida Liu, Liyun Zhang, Yan Zhang

**Affiliations:** ^1^Shanxi Bethune Hospital, Shanxi Academy of Medical Sciences, Third Hospital of Shanxi Medical University, Shanxi Province Clinical Research Center for Dermatologic and Immunologic Diseases (Rheumatic Diseases), Shanxi Province Clinical Theranostics Technology Innovation Center for Immunologic and Rheumatic Diseases, Taiyuan, China; ^2^Shanxi Academy of Advanced Research and Innovation (SAARI), Taiyuan, China; ^3^Laboratory of International Agro-Informatics, Department of Global Agricultural Sciences, Graduate School of Agricultural and Life Sciences, The University of Tokyo, Tokyo, Japan; ^4^Third Hospital of Shanxi Medical University, Shanxi Bethune Hospital, Shanxi Academy of Medical Sciences, Taiyuan, China; ^5^School of Public Health, Shanxi Medical University, Taiyuan, China; ^6^Second Clinical College, Shanxi University of Chinese Medicine, Jinzhong, China; ^7^National Institute for Nutrition and Health, Chinese Center for Disease Control and Prevention, Beijing, China

**Keywords:** dietary patterns, compositional data, hyperuricemia, principal component analysis, China health and nutrition survey

## Abstract

**Background/objectives:**

Dietary patterns play an important role in regulating serum uric acid (SUA) levels in the body. Recently, compositional data analysis (CoDA) has been proposed as an alternative technique in identifying dietary patterns. However, the relative advantages of CoDA, particularly in identifying dietary patterns associated with hyperuricemia have not been investigated. We evaluated and compared CoDA, including compositional principal component analysis (CPCA) and principal balances analysis (PBA), with the most commonly used principal component analysis (PCA) in determining dietary patterns associated with hyperuricemia.

**Methods:**

The 3 day 24-h dietary recall method was used to estimate dietary data from 3,954 study participants of the China Health and Nutrition Survey (CHNS). Dietary patterns were constructed using PCA, CPCA and PBA. These methods were compared based on the performance to identify plausible patterns associated with hyperuricemia.

**Results:**

PCA, CPCA and PBA all identified three dietary patterns, with a common “traditional southern Chinese” pattern high in rice and animal-based foods and low in wheat products and dairy. Only this pattern was positively associated with risk of hyperuricemia [PCA: OR (95%CI) = 1.29 (1.15–1.46); CPCA: OR (95%CI) = 1.25 (1.10–1.40); PBA: OR (95%CI) = 1.23 (1.09–1.38)].

**Conclusion:**

All three dietary patterns methods in our study identified that a “traditional southern Chinese” dietary pattern was associated with increased risk of hyperuricemia, suggesting a robust and consistent finding.

## 1 Introduction

Hyperuricemia, a metabolic condition characterized by excessive concentration of serum uric acid (SUA), poses a significant risk factor of the development of chronic diseases including gout, cardiovascular disease, and diabetes, etc. (1). Latest data shows that the prevalence of hyperuricemia in Chinese population has increased from 8.4% in 2010 to 13.6% in 2021 (2, 3), signaling the need for effective interventions to mitigate the rising rate of hyperuricemia. A growing body of research indicates that hyperuricemia can be effectively managed non-pharmacologically through dietary modifications such as avoiding alcohol consumption and adhering to a diet low in purine-rich foods (4–7). Additionally, plant-based foods, including vegetable, fruits, cereals, and spices, are rich in dietary polyphenols, which are shown to prevent hyperuricemia through inhibiting uric acid (UA) synthesis and enhancing UA secretion (8). Despite the potential benefits, studying human diets is largely complicated due to the impracticality of isolating diets into single foods or nutrients. Therefore, it is increasingly common in the field of nutrition epidemiology to investigate dietary patterns as a more holistic approach to capture day-to-day eating habits (9).

In most of the literature, statistical methods of deriving dietary patterns fall into three classic categories: *a priori*, *a posteriori*, and hybrid methods (9). The *a priori* approach, based on established knowledge and evidence, focuses on diet quality using various dietary indices, including Mediterranean scores and Healthy Eating Index, but tends to overlook the contributions of specific nutrients and the interactions between nutrients (10). In contrast, the *a posteriori* method is data-driven, extracting dietary patterns through statistical dimension-reduction techniques. The most widely used data-driven method is principal component analysis (PCA), which analyzes the correlation matrix of food variables and derives principal components (PC) or dietary patterns characterized by different foods (11). Hybrid methods combine the first two classes of methods to identify dietary patterns (9).

Over the past few years, a growing number of studies have emerged to use PCA as the primary method to investigate the impact of dietary patterns on hyperuricemia among Chinese adult populations, but the results have been inconsistent (12, 13). For example, while one cohort study found no association between a dietary pattern high in fruits, deep-fried foods, and sweets with hyperuricemia (14), another cohort study identified a similar pattern rich in sweet foods that was positively associated with hyperuricemia (1). Furthermore, when a cross-sectional study identified a plant-based dietary pattern inversely associated with hyperuricemia (13), another found no such association (15). A possible explanation for the discrepancies in these results is that PCA is not completely data-driven. When intending to qualitatively interpret the PC factors, the threshold value of foods’ factor loadings, coupled with the labeling of each dietary pattern, remains somewhat arbitrary (16). The inconsistencies in dietary patterns identified by PCA across studies suggest a potential weakness in using a single approach to derive patterns related to hyperuricemia.

Given that the amount of food intakes for an individual is relatively constant, the increase in the intake of some foods will lead to the decrease in the intake of other foods, vice versa. This implies the compositional nature of dietary intake, which can be adequately addressed by Compositional Data Analysis (CoDA) (9). CoDA is a novel class of dimension reduction methods that has not been widely utilized in health and dietary research (9). It encompasses a standard family of statistical methods computing log-ratio transformation of dietary data. These methods include compositional PC analysis (CPCA) and principal balances analysis (PBA), which serve as viable tools in estimating the relative importance of food variables within dietary patterns and facilitating the interpretation of the results (17).

Despite their promising application in dietary pattern research, limited studies have utilized these statistical methods (17). Moreover, the majority of studies have only chosen PCA as the single a posteriori approach to extract dietary patterns as hyperuricemia predictors, resulting in a lower reproducibility of the results (12). Therefore, the aim of this study is to (1) simultaneously use three statistical approaches – one traditional approach (i.e., PCA) and two novel approaches (i.e., PBA and CPCA) – to extract dietary patterns and explore shared food groups across these patterns; and (2) to investigate their associations with hyperuricemia in a cohort of Chinese population.

## 2 Materials and methods

### 2.1 Study design and population

This retrospective cohort study utilized data from the China Health and Nutrition Survey (CHNS), an ongoing large-scale, prospective cohort survey initiated in 1989 and continued in 1991, 1993, 1997, 2000, 2004, 2006, 2009, 2011, and 2015. Multi-stage random cluster sampling was employed to select the study participants from nine provinces and three autonomous cities with diverse demographic, geographical, economic development, and public resource characteristics. A comprehensive description of the CHNS is provided elsewhere (18). Given that the only hyperuricemia data available was in 2009, and the revised version of the China Food Composition Table (FCT) was initially used in 2004 to obtain nutrient values (19), we used data from the CHNS conducted in 2004, 2006, and 2009.

We included 14,086 participants who entered the study in either 2004 or 2006 and were followed up until 2009. We excluded 2,495 participants aged under 18 years and 4,853 who had missing hyperuricemia data. From the remaining participants, we further excluded pregnant women, those with diabetes, myocardial infarction, stroke, hypertension, and extreme total energy intake (≥ 8,000 and ≤ 800 kcal/day for men; ≥ 6,000 and ≤ 600 kcal/day for women) (16), and those who self-identified as vegetarians. Additionally, we excluded 912 participants with missing data on marital status, education, income, smoking, drinking, Body Mass Index (BMI), sleep, physical activity, and sedentary behavior (Supplementary Figure S1). Finally, a total sample of 3,954 participants were selected for the analysis.

### 2.2 Dietary assessment

To collect dietary data and assess individual diet, the CHNS employed a consecutive 3-day 24-h diet recall method. Literature has validated the use of the 24-h dietary recall method (20). Details of the dietary assessment has been described elsewhere (21). In summary, qualified interviewers requested that participants report the quantities and types of foods consumed on the preceding day for three consecutive days, which were randomly assigned from Monday to Sunday (21). We calculated the three-day average intake (grams per day) of foods for each participant in each survey round (2004, 2006, 2009) based on the FCT. We categorized 20 food groups according to their nutrient and culinary characteristics (see Supplementary Table S1).

### 2.3 Outcome measurement: SUA and hyperuricemia

The primary outcome of the study was hyperuricemia. In 2009, fasting blood samples (12 mL) were collected from participants. The SUA was measured using the enzymatic colorimetric method with a Hitachi 7,600 automated analyzer (Tokyo, Japan) and Randox reagents (Randox Laboratories Ltd., Crumlin, UK). Hyperuricemia was defined as SUA levels ≥ 416 μmol/L in men and ≥ 357 μmol/L in women (22).

### 2.4 Covariates

In line with the literature (22), the following confounders were included in the analysis due to their established association with hyperuricemia: age, sex, residence (urban and rural), region (southern and northern), marital status (married and others), education level (primary school and below, middle school, high school, college degree and above), income, hours of physical activity (PA), drinking, smoking, daily energy intake, BMI, sleep duration, and sedentary time. Smoking status was categorized as former smoker, current smoker, or non-smoker. Drinking status was classified into two categories: those who consumed beer/liquor in the previous year and those who did not. Anthropometric measurements including height and weight were taken using standard procedures, with participants wearing light clothing and no shoes. Body Mass Index (BMI) was calculated as weight in kilograms divided by height in meters squared (kg/m^2^), and was classified according to recommended cutoff points: underweight (<18.5), normal (18.5–23.9), overweight (24.0–27.9), and obese (≥28.0) (21). The total hours of PA and sedentary behavior were calculated based on the reported weekly hours of each activity type.

### 2.5 Statistical analysis

We obtained dietary patterns using three dimension reduction methods: PCA, PBA, and CPCA. Many of the 20 food groups included in the dietary analysis were consumed by only a small proportion of participants. For PCA, the food groups were classified into two categories: those consumed by <25% of participants were converted to binary variables (non-consumers and consumers), while those consumed by ≥25% of participants were categorized into three levels (non-consumers, consumers with intake ≤ median, and consumers with intake > median) (16). We used varimax orthogonal rotation to enhance the interpretability of factor correlations with food groups and retained factors with eigenvalues > 1.0 to maximize variance retention while reducing dimensionality. Each retained factor was interpreted based on factor loadings, reflecting correlations between food groups and the pattern. Patterns were labeled using food groups with absolute factor loadings≥0.3, indicating strong contributions.

CoDA uses isometric log-ratio transformations to generate new variables (dietary patterns) that represent log-ratios between compositional parts (17). To handle zero values from rarely consumed foods in the three-day 24-h dietary recalls, we applied zero imputation using a modified expectation–maximization algorithm with a lower detection limit (16). Consistent with CoDA principles, individual food group intakes were expressed as proportions of the total intake across all 20 groups. We applied PBA to generate principal balances (PBs) that quantify the relative contribution of specific food group subsets against another subset (16). Given the absence of a standardized criterion for PBs retention, we retained the same number of dietary patterns as derived from PCA. For the alternative CoDA approach, CPCA, we computed the log-ratio of each food group relative to the geometric mean of all 20 groups (17). Similar to PCA, each CPCA-derived PC involves all food groups, with absolute factor loadings≥0.3 indicating significant contributions to the pattern. The criterion for retaining PCs in CPCA mirrored that used in PCA.

Baseline characteristics were compared between participants with and without hyperuricemia, using chi-square tests for categorical variables and either Student’s *t*-tests or analysis of variance (ANOVA) for continuous variables, as appropriate. Tertiles for each dietary pattern were constructed to assess the associations between dietary patterns and the risk of hyperuricemia in three multivariate logistic regression models: Model 1 adjusted for age and sex; Model 2 additionally adjusted for marital status, residence, region, education, and income; Model 3 further adjusted for smoking, drinking, BMI, physical activity time, sedentary time, sleep duration, and daily energy intake.

All statistical analyses were performed using R version 4.0.3. The two-sided *p* < 0.05 was considered as statistical significance.

## 3 Results

### 3.1 Characteristics of study participants

After applying a series of exclusion criteria (see Supplementary Figure S1), a total of 3,954 participants were included in the cohort. Baseline characteristics of participants with and without hyperuricemia are summarized in Table 1. We documented 534 participants with hyperuricemia, with the overall prevalence of 13.5%. Compared to participants without hyperuricemia, those with hyperuricemia were more likely to be men, older, urban residents, southerners, highly educated, current smokers, current drinkers, and to have higher BMI, higher income, and shorter sleep duration.

**Table 1:** Baseline characteristics of the 3,954 study participants.

Characteristic	All participants (*n* 3,954)	Hyperuricemia		*p* value
		No (*n* 3,420)	Yes (*n* 534)	
Sex, *n* (%)				
Men	1827 (46.2)	1,487 (43.5)	340 (63.7)	<0.001
Women	2,127 (53.8)	1933 (56.5)	194 (36.3)	
Age, mean (SD), yr	45.7 (12.2)	45.5 (12.1)	47.2 (12.5)	0.003
Residence, *n* (%)				
Urban	1,124 (28.4)	930 (27.2)	194 (36.3)	<0.001
Rural	2,830 (71.6)	2,490 (72.8)	340 (63.7)	
Region, *n* (%)				
Northern	1,541 (39.0)	1,380 (40.4)	161 (30.2)	<0.001
Southern	2,413 (61.0)	2040 (59.7)	373 (69.9)	
Marriage status, *n* (%)				
Married	3,590 (90.8)	3,111 (91.0)	479 (89.7)	0.35
Unmarried	364 (9.2)	309 (9.0)	55 (10.3)	
Education, *n* (%)				
Primary school and below	1,665 (42.1)	1,457 (42.6)	208 (39.0)	0.001
Middle school	1,353 (34.2)	1,190 (34.8)	163 (30.5)	
High school	529 (13.4)	437 (12.8)	92 (17.2)	
College degree and above	407 (10.3)	336 (9.8)	71 (13.3)	
Smoking status, *n* (%)				
Non-smoker	2,676 (67.7)	2,379 (69.6)	297 (55.6)	<0.001
Former smoker	93 (2.4)	71 (2.1)	22 (4.1)	
Current smoker	1,185 (30.0)	970 (28.4)	215 (40.3)	
Drinking status, *n* (%)				
Never	2,642 (66.8)	2,363 (69.1)	279 (52.3)	<0.001
Current	1,312 (33.2)	1,057 (30.9)	255 (47.8)	
BMI, *n* (%)				
Underweight	238 (6.0)	223 (0.03)	15 (2.8)	<0.001
Normal	2,448 (61.9)	2,188 (64.0)	260 (48.7)	
Overweight	1,051 (26.6)	848 (24.8)	203 (38.0)	
Obese	217 (5.5)	161 (4.7)	56 (10.5)	
Income, mean (SD), CNY/yr	10289.3 (13675.3)	9958.5 (13332.7)	12407.7 (15541.0)	<0.001
Sleep duration, mean (SD), h/d	8.1 (1.2)	8.1 (1.2)	8.0 (1.2)	0.04
Physical activity time, mean (SD), h/d	0.4 (1.8)	0.4 (1.9)	0.41 (1.3)	0.97
Sedentary time, mean (SD), h/d	4.9 (3.94)	4.9 (4.0)	4.91 (3.6)	0.76
Energy, mean (SD), kcal/d	2282.4 (660.0)	2275.8 (657.5)	2324.40 (674.9)	0.11
Serum uric acid, mean (SD), μmol/L	301.1 (102.2)	273.8 (66.0)	476.42 (118.3)	<0.001

BMI, Body Mass Index; CNY/yr, Chinese yuan/year; SD, standard deviation.

### 3.2 Dietary patterns based on PCA, PBA, and CPCA

Factor loadings of the 20 food groups for the dietary patterns derived from PCA, PBA, and CPCA are shown in Table 2. PCA identified three distinct dietary patterns, which were determined by three PC factors with eigenvalues greater than 1.0. The first PC factor (“traditional southern Chinese”) had low intakes of wheat, milk, and other cereals, with high intakes of fresh vegetables, pork, aquatic products, and rice. The second PC factor (“western”) had high intakes of fast foods, sugary food, fruits, beverages, eggs, processed meat, and fungi and algae. The third PC factor (“plant-based”) was characterized by high intakes of tubers, legumes, and fresh vegetables, and low intakes of other livestock and organs. The three PCs explained 28.07% of the total variation.

**Table 2:** Factor loadings of for patterns derived using principal component analysis (PCA), principal balances analysis (PBA), and compositional principal component analysis (CPCA).

Food groups	PCA			PBA			CPCA		
	Factor 1	Factor 2	Factor 3	Factor 1	Factor 2	Factor 3	Factor 1	Factor 2	Factor3
Wheat products	**−0.78**	−0.02	0.25	**−0.44**	**−0.41**		**−0.83**	−0.12	−0.14
Dairy	−**0.76**	0.03	0.22	**−0.44**	**−0.41**		**−0.83**	−0.11	−0.10
Tubers	−0.08	0.00	**0.53**	**−0.44**	**0.82**		−0.22	−0.09	**−0.56**
Other cereals	−**0**.**56**	−0.02	−0.13			**−0.82**	−0.08	0.09	−0.10
Fungi and algae	0.07	**0.45**	−0.19				−0.04	−0.02	**0.51**
Eggs	0.04	**0.33**	0.24			**0.41**	−0.02	−0.29	−0.14
Beverages	0.02	**0.31**	−0.04				−0.02	**0.92**	0.05
Fruits	0.02	**0.66**	−0.01			**0.41**	−0.00	−0.24	0.21
Vegetables	**0.31**	−0.14	**0.31**				0.01	**0.91**	−0.06
Fast Foods	−0.04	**0.56**	0.06				0.07	0.08	−0.12
Sweets	−0.04	**0.46**	0.09				0.08	0.11	−0.05
Other livestock meat	0.06	0.14	**−0.43**				0.10	0.02	−0.08
Organs	0.06	0.05	**−0.50**				0.10	**0.30**	0.11
Processed meat	0.04	**0.32**	−0.17				0.12	**0.39**	−0.11
Poultry	0.16	0.29	−**0.42**	**0.33**			0.15	−0.01	**0.62**
Nuts	−0.00	0.25	−0.11				0.20	0.24	0.07
Rice	**0.63**	−0.27	0.06	**0.33**			**0.33**	**0.32**	0.04
Aquatic products	**0.35**	0.11	0.01	**0.33**			**0.36**	−0.13	**0.41**
Legumes	0.10	0.17	**0.48**				**0.37**	−0.30	**−0.40**
Pork	**0.34**	0.17	−0.09	**0.33**			**0.45**	−0.15	0.14
Variance explained (%)	12.10	9.46	6.51	16.84	9.14	8.85	13.51	11.51	6.98

Bold values indicate food groups that were significant contributors to the patterns (with factor loadings ≥ |0.30|).

PBA analysis also identified three dietary patterns. The first PB (“traditional southern Chinese”) showed high intakes of rice, poultry, aquatic products, and pork, with low intakes of wheat, tubers, and milk. The second PB (“Tuber-Based”) had high intakes of tubers and low intakes of milk and wheat. The third PB (“low cereal”) was characterized by high intakes of fruits and eggs and a low intake of other cereals. The three PBs accounted for 34.83% of the food intake variations.

Similar to the first PC and the first PB, the CPCA presents that the first PC (“traditional southern Chinese”) was characterized by high intakes of rice, aquatic product, legumes, and pork and low intakes of wheat and milk. The second PC (“mixed”) had high intakes of beverages, vegetables, organ, processed meat, and rice. The third PC (“seafood and poultry”) showed high intakes of fungi and algae, poultry, aquatic products, with low intakes of tubers and legumes. The total variance explained by the three PCs was 32%.

### 3.3 Dietary patterns and hyperuricemia

Table 3 displays the association between the risk of hyperuricemia with dietary patterns obtained from PCA, PBA and CPCA. For PCA, after adjusting for all covariates (in Model 3), the “traditional southern Chinese” pattern was positively associated with hyperuricemia (adjusted OR for T3 vs. T1, 1.68; 95% CI, 1.31–2.14; *P* trend < 0.001), while the “plant-based” pattern was inversely associated with hyperuricemia (adjusted OR for T3 vs. T1, 0.76; 95% CI, 0.60–0.96; *P* trend = 0.02). Similarly, the “traditional southern Chinese” pattern derived from PBA was positively associated with hyperuricemia (adjusted OR for T3 vs. T1, 1.43; 95% CI, 1.12–1.84; *P* trend = 0.001), while the other two patterns of PBA showed no association with hyperuricemia. The “traditional southern Chinese” pattern derived from CPCA was also positively associated with hyperuricemia (adjusted OR for T3 vs. T1, 1.57; 95% CI, 1.23–2.01; *P* trend < 0.001).

**Table 3:** Odds ratios (95% CI) for risk of hyperuricemia by tertile of each dietary pattern scores derived from principal component analysis (PCA), principal balances analysis (PBA), and compositional principal component analysis (CPCA).

Models	T1	T2	T3	*P*-trend
		OR (95%CI)	OR (95%CI)	
Principal component analysis				
*PC1: “traditional southern Chinese” pattern*				
Model 1	Ref.	1.41 (1.11, 1.78)	1.43 (1.13, 1.81)	0.002
Model 2	Ref.	1.37 (1.08, 1.74)	1.42 (1.12, 1.79)	0.003
Model 3	Ref.	1.54 (1.21, 1.97)	1.68 (1.31, 2.14)	<0.001
*PC2: “western” pattern*				
Model 1	Ref.	1.20 (0.95, 1.51)	1.15 (0.91, 1.45)	0.26
Model 2	Ref.	1.12 (0.89, 1.41)	0.94 (0.73, 1.21)	0.61
Model 3	Ref.	1.06 (0.83, 1.34)	0.90 (0.69, 1.16)	0.40
*PC3: “plant-based” pattern*				
Model 1	Ref.	0.77 (0.62, 0.97)	0.76 (0.60, 0.95)	0.01
Model 2	Ref.	0.84 (0.67, 1.05)	0.77 (0.61, 0.97)	0.02
Model 3	Ref.	0.80 (0.63, 1.02)	0.76 (0.60, 0.96)	0.02
Principal balances				
*PB1: “traditional southern Chinese” pattern*				
Model 1	Ref.	1.11 (0.88, 1.40)	1.37 (1.08, 1.74)	0.005
Model 2	Ref.	1.17 (0.93, 1.48)	1.29 (1.01, 1.64)	0.02
Model 3	Ref.	1.30 (1.02, 1.65)	1.43 (1.12, 1.84)	0.001
*PB2: “tuber-based” pattern*				
Model 1	Ref.	1.01 (0.81, 1.27)	1.02 (0.81, 1.28)	0.93
Model 2	Ref.	1.07 (0.85, 1.34)	1.08 (0.86, 1.36)	0.54
Model 3	Ref.	1.06 (0.84, 1.33)	1.08 (0.85, 1.36)	0.55
*PB3: “low cereal” pattern*				
Model 1	Ref.	1.13 (0.89, 1.44)	1.06 (0.84, 1.35)	0.46
Model 2	Ref.	1.19 (0.93, 1.51)	1.05 (0.83, 1.34)	0.68
Model 3	Ref.	1.21 (0.94, 1.55)	1.03 (0.81, 1.31)	0.91
Compositional PCA				
*PC1: “traditional southern Chinese” pattern*				
Model 1	Ref.	0.97 (0.78, 1.20)	1.48 (1.17, 1.88)	0.002
Model 2	Ref.	0.99 (0.79, 1.23)	1.40 (1.10, 1.78)	0.01
Model 3	Ref.	1.09 (0.87, 1.36)	1.57 (1.23, 2.01)	< 0.001
*PC2: “mixed” pattern*				
Model 1	Ref.	1.12 (0.89, 1.40)	1.11 (0.88, 1.39)	0.40
Model 2	Ref.	1.12 (0.89, 1.40)	1.08 (0.86, 1.36)	0.49
Model 3	Ref.	1.14 (0.90, 1.44)	1.12 (0.89, 1.42)	0.32
*PC3: “seafood and poultry” pattern*				
Model 1	Ref.	0.88 (0.70, 1.10)	1.13 (0.89, 1.43)	0.49
Model 2	Ref.	0.93 (0.74, 1.17)	1.24 (0.97, 1.57)	0.12
Model 3	Ref.	0.94 (0.75, 1.18)	1.26 (0.98, 1.60)	0.10

Results were obtained from multivariate logistic regression models; Model 1: adjusted for age and sex; Model 2: additionally adjusted for marital status, region, residence, education, and income; Model 3, further adjusted for smoking, drinking, BMI, physical activity time, sedentary time, sleep duration, and daily energy intake. PC, principal component; PB, principal balance; OR, odds ratios; CI, confidence interval; T, tertile.

## 4 Discussion

This study applied three analytical methods—PCA and two data-driven CoDA methods (PBA and CPCA)—each identified three dietary patterns in a cohort of 3,954 Chinese adult participants. The three methods harmonized in identifying a “traditional southern Chinese” dietary pattern with shared attributes: lower wheat and dairy intake, higher rice, aquatic, and pork intake, and a positive association with hyperuricemia risk. The high comparability suggests that methods focusing on food composition can effectively capture dietary behavior. Consistent findings across methods strengthen our understanding that a high-animal-product, low-dairy-and-cereal-product dietary pattern may increase hyperuricemia risk among Chinese.

Evidence shows that traditional agricultural practices in southern China have led to a dominant rice-based dietary pattern (23). With the rapid aquaculture development (24) and economic growth especially in the southern coastal region in China, the traditional Chinese diet has transitioned towards a western-like diet with more animal-based foods including meats and seafood products (25). These products tend to have a high purine content, which elevates blood levels of uric acid, or urate (the end product of purine), making it one of the major risk factors for hyperuricemia (26). The adverse effect of animal meats, organs, and seafoods on the outcome of hyperuricemia as well as disorders like gout has been confirmed in various studies (27–30). Research supports our observation that hyperuricemia was prevalent among male participants with higher incomes living in economically developed urban southern Chinese regions, likely due to frequent purine-rich diet consumption (30, 31).

Literature suggests that plant-based foods like wheat and cereals are beneficial for lowering SUA and the risk of hyperuricemia because they contain minimal purines (26, 32). Not all animal-based foods increase the risk of hyperuricemia. Dairy products, like milk and cheese, are also linked to reduced uric acid levels (26, 28). A cohort study found a strong inverse relationship between dairy products and gout, suggesting dairy proteins and other nutrients in dairy products cause urate lowering (28). Increased consumption of urate-elevating foods (like meat and seafood) and decreased consumption of urate-lowering foods (like wheat and dairy) may explain the positive association between the “traditional southern Chinese” pattern and hyperuricemia risk in this study.

Another cohort study of Chinese adults identified a “traditional southern” dietary pattern using PCA, similar to ours. This pattern is characterized by low whole grain and cereal intake and high pork, fish, offal, and poultry intake. It was also positively associated with the risk of hyperuricemia in a full adjusted model (13). In contrast, we found a significant inverse association between the “plant-based” pattern and hyperuricemia, similar to a China-based study by Zhou et al., though the food group compositions differed (13). Our “plant-based” pattern emphasized legumes, tubers, and vegetables, while theirs included legumes, vegetables, and non-plant-based foods like eggs and dairy (13). Similarly, using PCA, Mao et al. identified a plant-based dietary pattern high in tuber and vegetables, which had a reverse association with the SUA levels (33). Moreover, a comprehensive meta-analysis study supported our findings, showing a positive association between animal-based diets and hyperuricemia, while plant-based diets showed an inverse relationship (34).

Previous studies defining dietary patterns using multiple methods yielded inconclusive results regarding their correlation with hyperuricemia risk (12). For instance, in the cohort study by Zhou et al., different methods like PCA derived incomparable dietary patterns among northern Chinese adults, with varying associations with hyperuricemia (13). Due to inconsistent findings in the literature, it’s challenging to definitively establish dietary patterns associated with hyperuricemia. This study identified a dietary pattern significantly linked to hyperuricemia, replicated across three methods: conventional PCA and two novel CoDA methods. It is important to note that the “traditional southern Chinese” dietary pattern identified in our study differs in composition from the commonly referenced “traditional Chinese” or “eastern” dietary pattern (12). While the traditional Chinese diet is predominantly plant-based and characterized by lower meat consumption (33), the “traditional southern Chinese” pattern observed in our analysis is distinguished by having higher intakes of animal-based foods and lower intakes of dairy and cereal products. Therefore, in daily life, one should consciously increase the intake of plant-based foods and dairy products and reduce the intake of animal-based foods.

From a statistical perspective, these three methods differ in their methodological approaches, each with its own strengths and limitations. PCA, a commonly used technique, constructs factors based on variance in food group intake to capture dietary patterns (35). However, it lacks a standard threshold value of factor loadings, introduces subjectivity in pattern naming, and cannot model food substitution effects (16). CoDA methods (PBA and CPCA), in contrast, model substitution by representing dietary patterns as balances between food groups (17). PBA simplifies pattern naming by focusing on a smaller number of food groups but also lacks standard retention rules, whereas CPCA, similar to PCA, presents all food groups but retains subjectivity in labeling. Despite these methodological differences, the results from all three approaches converged, consistently identifying an association between the “traditional southern Chinese” dietary pattern and hyperuricemia in the Chinese population. This convergence strengthens the robustness of our conclusion.

This study has several strengths. To the best of our knowledge, it is the first to use the recently developed CoDA methods to find dietary patterns linked to hyperuricemia in a Chinese population over time. The large sample size and careful control of covariates enhanced the findings’ reliability. Consistent identification of a similar dietary pattern associated with hyperuricemia across three methods demonstrated reproducibility. However, this study has several limitations. First, we were unable to account for unobserved potential confounders, including possible impacts of drug use (such as diuretics) or genetic factors (such as the SLC2A9 genotype) on UA (36), thereby establishing causality remains challenging, as is the case with all observational studies. Additionally, although dietary data were collected repeatedly using an extensive database, the three-day dietary records may not fully capture individuals’ habitual eating patterns. Third, the generalizability of the results to other study settings, including different population groups and health outcomes, is questionable due to the current study population’s limited scope to general Chinese adults. Finally, the findings may not capture shifts in dietary patterns since the data used in this analysis were limited to 2009, the most recent year for which biomarker data were available. Future studies with more up-to-date data are warranted to validate and extend these findings.

## 5 Conclusion

In conclusion, we extract dietary patterns through three data-driven methods and explored the relationships between the dietary patterns and the risk of hyperuricemia. All three methods – PCA, PBA, and CPCA – consistently identified one common dietary pattern that was positively associated with the risk of hyperuricemia: the “traditional southern Chinese” pattern, characterized by a high intake of rice and animal-based foods and a low intake of wheat products and dairy. On the other hand, the “plant-based” pattern may also be inversely associated with hyperuricemia, but further verification is still needed, while the other dietary patterns showed no association with hyperuricemia. The findings indicate that reducing animal-based food consumption and increasing the intake of wheat products and dairy, as “eastern” dietary pattern advocated, may be advantageous in preventing hyperuricemia. Future longitudinal studies conducted in other settings are necessary to validate our results and explore causal mechanisms.

## Data Availability

Publicly available datasets were analyzed in this study. This data can be found at: http://www.cpc.unc.edu/projects/china.
