# Supplementary material for: Comparison between compositional data analysis and principal component analysis for identifying dietary patterns associated with hyperuricemia
Source: Front Nutr. 2025 Jul 16;12:1582674. doi: 10.3389/fnut.2025.1582674 (PMC12307145; doi:10.3389/fnut.2025.1582674)
Supplement: Supplementary file 1 [file Table_1.docx]

Supplementary material


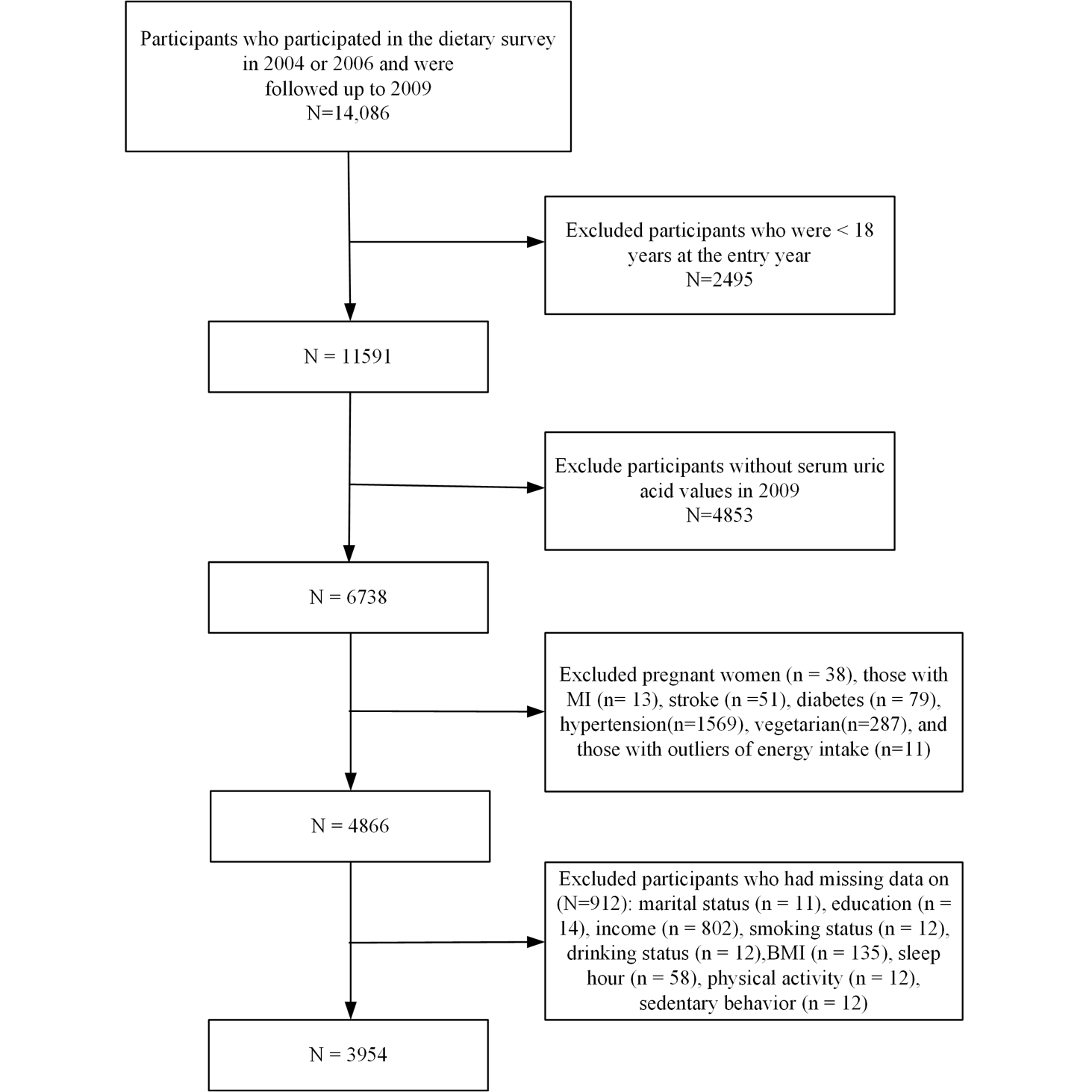


Figure S1. Flowchart of sample selection

**Table S1.** Examples of food items for each food group

| **Food groups** | **Examples of food items** |
| --- | --- |
| Rice | Round-grained rice, long-grained rice, glutinous rice |
| Wheat | Wheat bun, wheat noodles |
| Other cereals | Maize, barley, millet |
| Tubers | Potato, sweet potato |
| Legumes | Soyabeans, and products |
| Fungi and algae | Mushroom, kelp, laver |
| Vegetables | Cabbage, eggplant, carrot, pepper, lettuce, rape, tomato, cauliflower |
| Fruits | Apple, pear, peach, date, grape, watermelon, orange, other fruit |
| Pork | Pork and pork products |
| Other livestock meat | Beef, game, lamb, meat products |
| Poultry | Chicken, duck, goose |
| Organ meat | Organ meat |
| Processed meat | Sausages, ham, luncheon meat, dried meat, smoked meat |
| Aquatic products | Fish, shrimp, crab, shellfish |
| Milk | Milk and products |
| Eggs | Eggs |
| Nuts | Nuts |
| Sugary foods | Jelly, jam, chocolate, honey, sugar, candies |
| Fast foods | Convenience food, hamburger, pizza, sandwich, French fries |
| Beverages | Fruit or flavoured drinks, fruit juice, soft drink |
